# Pediatric Local Diagnostic Reference Levels for CT Examinations: A Study Based on the European Guidelines on Diagnostic Reference Levels for Pediatric Imaging

**DOI:** 10.7759/cureus.85284

**Published:** 2025-06-03

**Authors:** Vasilis Syrgiamiotis, Agapi Ploussi, Stamatis Rallis, Maria M Gavra, Efthimia Alexopoulou, Kalliopi Platoni, Efstathios P Efstathopoulos

**Affiliations:** 1 2nd Department of Radiology, Medical School, Attikon University Hospital, National and Kapodistrian University of Athens, Athens, GRC; 2 Department of Pediatric Radiology (CT, MRI, PET/CT), Aghia Sophia General Children’s Hospital, Athens, GRC; 3 2nd Department of Radiology, Medical School, Attikon University Hospital, National and Kapodistrian University, Athens, GRC

**Keywords:** dose reference level, european guidelines, local diagnostic reference level, pediatric body ct weight grouping, pediatric computed tomography, pediatric head ct age grouping, pediatric population, pediatric radiology, pediatric research, radiation dose reduction

## Abstract

Background: Diagnostic reference levels (DRLs) are valuable tools for computed tomography (CT) dose optimization. The current study aimed to establish local DRLs (LDRLs) in pediatric patients who underwent head, chest, and abdomen-pelvis (AP) CT examinations after implementing age- and weight-based protocols according to the European Guidelines on Diagnostic Reference Levels for Pediatric Imaging.

Methods: The study included a total of 357 pediatric patients in a 16-slice CT unit at the nation's largest pediatric hospital who underwent head, chest, and AP CT examinations within a six-month period, from winter 2023 to spring 2024. The CT protocols were categorized based on age for head CT examinations and weight for body CT examinations. Patients’ demographic characteristics and exposure parameters were recorded. In addition to age and body weight, no contour, anteroposterior, or lateral dimensions were taken into account. We were unable to automatically incorporate size-specific dose estimation (SSDE) into our research because our device did not supply it. DRLs were calculated based on the third quartile of the distribution (75^th^ percentile) of volume CT dose index (CTDIvol) and dose length product (DLP).

Results: LDRLs in terms of CTDIvol for head, chest, and AP CT examinations ranged from 15 to 65 mGy, 1 to 5 mGy, and 1 to 7 mGy, respectively. In terms of DLP per single acquisition, LDRLs for head, chest, and AP CT examinations varied from 211 to 787 mGy·cm, 18 to 226 mGy·cm, and 36 to 438 mGy·cm for the various patient groups, respectively. The proposed pediatric DRLs are lower than the European DRLs (EDRLs) except for patients above six years old in head CT examinations and the 50-80 kg weight category in chest CT examinations.

Discussion: We adjusted our age-based CT procedures in the current study to comply with the new European recommendations, Radiation Protection No. 185 (RP 185), which recommends weight groups for body CT examinations and particular age groups for head CT examinations. Following these modifications, we utilized the newly established patient groups to gather the dosimetric data (CTDIvol and DLP) in order to determine LDRLs for pediatric patients who had CT exams of the head, chest, and AP. To the best of our knowledge, the suggested LDRLs are the first nationwide attempt, in accordance with pediatric DRLs' age-weight-based methodologies, and constitute a baseline for national DRLs (NDRLs), which will be decided by this country's authorized authority. It sets the baseline for regular clinical audits within the department and encourages other facilities to do the same to standardize CT pediatric procedures. Currently, there are no comparisons throughout the entire nation. At the European level, it seems that a lot of work needs to be done to meet the new requirements.

Conclusion: The newly weight-based protocols for body CT examinations provided lower radiation doses than the age-based protocols, better reflecting the wide range of pediatric patients. Further CT optimization is needed for the heavier patients (50-80 kg).

## Introduction

Over the last decades, advances in computed tomography (CT) detectors and image reconstruction techniques have significantly increased the number of CT examinations worldwide. This increase is also reflected in pediatric CT imaging, which, according to United Nations Scientific Committee on the Effects of Atomic Radiation (UNSCEAR), represents over 10% of the total CT examinations [1].

It is well known that exposure to ionizing radiation can lead to carcinogenesis, especially when radiosensitive tissues are exposed to increased doses [2]. Children are more sensitive to radiation than adults for specific types of cancer, including brain, skin, breast, thyroid, and leukemia [3]. In addition, owing to their longer life expectancy, they have an increased risk of developing adverse radiation effects. To minimize the risk, CT protocols should be optimized to keep radiation doses "as low as reasonably achievable (ALARA) [4].

Diagnostic reference levels (DRLs) have been proven to be a valuable tool for monitoring optimization strategies in medical imaging [5]. DRLs are described as "a type of investigation level, utilized as a tool of the process of optimizing the procedure of protecting the exposure of patients for diagnostic and interventional procedures. DRLs are used in medical imaging with ionizing radiation to indicate whether, in routine conditions, the amount of radiation used for a specified procedure is unusually high or low for that procedure" [6]. They can be determined at a regional (local DRLs (LDRLs)), national (national DRLs (NDRLs)), or European (European DRLs (EDRLs)) level. For CT examinations, the recommended DRL quantities are both volume computed tomography dose index (CTDIvol) and dose length product (DLP) [6].

The nation's legislation defines the pediatric population as any person between the ages of one and 16, male or female, of varying weight, with or without symptoms, findings, or therapy, and who may be receiving follow-up care or only a single examination. This makes it difficult to establish pediatric DRLs, primarily because of the wide range of body sizes in the pediatric population and the lack of standardization in the patient grouping [7]. Recognizing the need to provide a consistent methodology for establishing pediatric DRLs, the European Commission, in 2018, published the European Guidelines on Diagnostic Reference Levels for Pediatric Imaging (Radiation Protection No 185 (RP 185)) [7]. Owing to the large variations in body size, even for children of the same age, the RP 185 recommended weight-based groups for all body examinations and age-based groups for head examinations [7].

Following the new guidelines, the age-band protocols of our department were modified according to the recommended patient grouping. The study aimed to establish pediatric LDRLs for head, chest, and abdomen-pelvis (AP) CT examinations after adopting the age- and weight-based CT protocols suggested by RP 185. Quality control accuracy (QA), patient sampling (PS), and data collection (DC) are the three pillars upon which our study has been built in order to ascertain LDRLs [8].

## Materials and methods

Patient population

The retrospective study consisted of 357 pediatric patients (under 16 years old) who underwent head, chest, and AP CT examinations in a dedicated children's hospital. The age-based CT protocols used at our department for both head and body examinations were modified and optimized according to RP 185. More specifically, the pediatric population was categorized into four age groups for head CT examinations (0 to three months, three months to one year, one to six years, and > six years) and into five weight groups for body CT examinations (<5 kg, 5 kg-<15 kg, 15 kg-30 kg, 30 kg-50 kg, and 50 kg-80 kg). Patients’ collected data included gender, age, and weight. The study was conducted in accordance with the principles outlined in the Declaration of Helsinki and was approved by the Bioethics and Ethics Committee of the General University Hospital of Athens "Attikon," Athens, Greece (approval number: 1255/25-1-16). 

CT data acquisition

All CT examinations were conducted on a 16-slice multidetector CT (MDCT) (Brightspeed, GE Healthcare, Milwaukee, WI, USA) equipped with automatic exposure control (AEC) and an iterative reconstruction (IR) algorithm (Adaptive statistical IR (ASiR), GE Healthcare, Chicago, IL, USA). Head CT scans were performed using a 2.5 mm slice thickness from the skull base (including foramen magnum) to the cerebellum tentorium and a 5 mm slice thickness from the cerebellum tentorium to the vertex. Body CT examinations were performed using a combined x-y-z axis automatic tube current modulation technique. For chest CT examinations, the scan region was limited from the thoracic inlet to the costophrenic angle, where the diaphragm and chest wall connect, whereas AP CT examinations covered the area from the thoracic diaphragm to the pelvic outlet. For each type of CT examination, scan mode, tube voltage, tube current, slice thickness, rotation time, noise index, ASiR level, and collimation were recorded. QA, PS, and DC are the three pillars upon which our study has been built in order to ascertain LDRLs.

Radiation dose assessment

Radiation exposure parameters like CTDIvol and DLP were obtained from the Picture Archiving and Communication System (PACS). In clinical practice, the department chief medical physicist has regularly calibrated or validated the values used for each beam quality in the Digital Imaging and Communications in Medicine (DICOM) header, at the display unit, and for patient dosage monitoring within tolerance levels. Occasionally, it occurs once every two months unless there is a malfunction. CTDIvol was based on a 16-cm-diameter phantom for head and on a 32-cm-diameter phantom for body CT examinations. LDRLs were calculated as the 75^th^ percentile of CTDIvol and DLP per single sequence. The median values of CTDIvol and DPL were also calculated for optimization purposes. The licensed software IBM SPSS Statistics software, version 26 (IBM Corp., Armonk, NY, USA), via the university's VPN, and Microsoft Excel for Mac, version 2018 (Microsoft Corp., Redmond, WA, USA), were used to calculate the 75th percentile values and median values (50^th^ percentile) as well. 

## Results

An overview of the patient characteristics data gathered from winter 2023 to spring 2024, including daily routine examinations from six different radiographers with varying levels of experience, with the exception of patients from the hospital's acute unit clinic and those who worked nights, weekends, and national holidays, is presented in Table 1 and Table 2. The mean age of the pediatric population was 6.6±1.6 years, and the weight was 25.3±4.5 kg. Most patients were male (58%). The most common examinations were head CT (64%), followed by chest (23%) and AP CT (13%). No data were provided in the first weight group of AP CT examination (<5 kg) since the sample size was less than 10 patients.

**Table 1:** Patient characteristics for head CT examination *Number of patients; age and weight are given as mean value±standard deviation; m: months; y: years

CT examination	Age group	N*	Male	Female	Age (y)	Weight (kg)
Head	0 – 3m	21	8	13	0.2±0.1	4.4±2.2
	3m – 1y	26	15	11	0.6±0.2	6.3±3.0
	1y – 6y	49	35	14	3.4±1.51	15.3±6.4
	>6y	134	73	61	10.5±2.6	35.7±13.6

**Table 2:** Patient characteristics for chest and abdomen-pelvis CT examination *Number of patients; age and weight are given as mean value±standard deviation; N/A: not available

CT examination	Weight group	N*	Male	Female	Age (y)	Weight (kg)
Chest	<5kg	15	12	3	0.3±0.2	3.0±1.3
	5kg-<15kg	14	10	4	2.3±0.6	11.6±1.0
	15kg-<30kg	13	10	3	6.3±2.7	18.8±2.3
	30kg-<50kg	18	7	11	11.6±4.4	38.6±5.6
	50kg-<80kg	21	13	8	13.9±3.5	62.0±9.1
Abdomen-pelvis	<5kg	N/A	N/A	N/A	N/A	N/A
	5kg-<15kg	13	7	6	1.7±0.7	10.5±2.1
	15kg-<30kg	10	7	3	10.2±0.6	26.6±2.6
	30kg-<50kg	12	7	5	10.6±1.6	39.1±2.1
	50kg-<80kg	11	3	8	14.6±2.3	57.8±6.9

CT data acquisition

Scan acquisition parameters are presented in Table 3 and Table 4. Head CT examinations were performed in axial mode, whereas body CT examinations were performed in helical mode.

**Table 3:** Acquisition parameters for head CT examination kVp: kilovoltage peak, tube voltage; mAs: milliampere-seconds, tube current values are given as a range; Sth: slice thickness; Rt: rotation time; NI: noise index; ASiR: adaptive statistical iterative reconstruction; Col: collimation; m: months; y: years; Reg a: head slice thickness region a, from the skull base to the tentorium of the cerebellum; Reg b: head slice thickness region b, from the tentorium of the cerebellum to the vertex

CT exam	Age group	kVp	mAs		Sth (mm)		Rt(s)	NI		ASiR	Col (mm)
			Reg a	Reg b	Reg a	Reg b		Reg a	Reg b		
Head	0 – 3m	80	239-290	230-232	2.5	5.0	1	3,80	2,80	50%	16 × 1.25
	3m –1y	80	179-290	177-260	2.5	5.0	1	3,80	2,80	50%	16 × 1.25
	1y – 6y	100	250-310	144-310	2.5	5.0	1	3,8	2,80	50%	16 × 1.25
	>6y	120	186-354	181-435	2.5	5.0	1	3,80	2,80	50%	16 × 1.25

**Table 4:** Acquisition parameters for chest and abdomen-pelvis CT examination kVp: kilovoltage peak, tube voltage; mAs: milliampere-seconds, tube current values are given as a range; Sth: slice thickness; Rt: rotation time; NI: noise index; ASiR: adaptive statistical iterative reconstruction; Col: collimation; N/A: not available

CT exam	Weight group	kVp	mAs	Sth (mm)	Rt(s)	NI	ASiR	Col (mm)
Chest	<5kg	80	24-43	2.5	0,6	13.48	40%	16 × 1.25
	5kg-15kg	100	30-37	2.5	0,6	14.27	40%	16 × 1.25
	15kg-30kg	100	25-51	3.75	0,7	11.12	40%	16 × 1.25
	30kg-50kg	120	28-52	5.0	0,8	10.41	40%	16 × 1.25
	50kg-80kg	120	40-156	5.0	0,8	10.41	40%	16 × 1.25
Abdomen-pelvis	<5kg	80	N/A	N/A	0,7	18.23	50%	16 × 1.25
	5kg-15kg	100	25-45	3.75	0,7	16.84	50%	16 × 1.25
	15kg-30kg	120	34-36	3.75	0,5	12.30	50%	16 × 1.25
	30kg-50kg	120	39-55	5.0	0,6	15.62	50%	16 × 1.25
	50kg-80kg	120	97-115	5.0	0,6	12.15	50%	16 × 1.25

Radiation dose assessment

Table 5 presents the median and 75^th^ percentile values of CTDIvol and DLP per acquisition for head age group CT examinations, and Table 6 presents the median and 75^th^ percentile values of CTDIvol and DLP for chest and AP weight group CT examinations.

**Table 5:** Median and 75th percentile of CTDIvol and DLP per acquisition for head CT examination CTDIvol: volume computed tomography dose index; DLP: dose length product; CTDIvol region a: using a slice thickness of 2.5 mm from the skull base to the tentorium of the cerebellum; CTDIvol region b: using a slice thickness of 5.0 mm from the tentorium of the cerebellum to the vertex; †DLP concerns a single sequence; m: months; y: years

CT examination	Age group	N*	Median				75^th^ percentile			
			CTDIvol (mGy)			DLP†(mGy·cm)	CTDIvol (mGy)			DLP†(mGy·cm)
			region a	region b		single sequence	region a	region b		
Head	0– 3m	21	18		15	152	21		15	211
	3m – 1y	26	19		15	212	22		16	251
	1y – 6y	49	44		24	412	45		27	476
	>6y	134	59		40	660	65		45	787

**Table 6:** Median and 75th percentile of CTDIvol and DLP per acquisition for chest and abdomen-pelvis CT examination CTDIvol: volume computed tomography dose index; DLP: dose length product; ^†^DLP concerns a single sequence; N/A: not available; N*: number of patients

CT examination	Weight group	N*	Median		75^th^ percentile	
			CTDIvol (mGy)	DLP†(mGy·cm)	CTDIvol (mGy)	DLP†(mGy·cm)
Chest	<5kg	15	1	13	1	18
	5kg-<15kg	14	1	31	2	33
	15kg-<30kg	13	2	40	2	42
	30kg-<50kg	18	3	71	3	74
	50kg-<80kg	21	5	166	7	226
Abdomen-pelvis	<5kg	N/A	N/A	N/A	N/A	N/A
	5kg -<15kg	13	1	33	1	36
	15kg-<30kg	10	2	59	2	63
	30kg -<50kg	12	3	89	3	89
	50kg –<80kg	11	7	324	7	438

Table 7 and Table 8 show the DRL values obtained from the present study in comparison to the EDRLs and a previous study conducted by the authors that utilized age-based protocols [9].

**Table 7:** 75th percentile of CTDIvol and DLP per acquisition for head CT examination according to age , compared with the EDRLs and the values from our previous study. CTDIvol: volume computed tomography dose index; DLP: dose length product; EDRL: European diagnostic reference levels; CTDIvol region a: using a slice thickness of 2.5 mm from the skull base to the tentorium of the cerebellum; CTDIvol region b: using a slice thickness of 5.0 mm from the tentorium of the cerebellum to the vertex; †DLP concerns a single sequence; m: months; y: years; N/A: not available; CTDIvol in mGy; DLP in mGy·cm

CT examination	Age group	Current study			EDRLs		Authors’ previous study [9]		
		CTDIvol		DLP†	CTDIvol	DLP†	CTDIvol		DLP†
		region a	region b				region a	region b	
Head	0 – 3 m	21	15	211	24	300	27	22	317
	3m – 1y	22	16	251	28	385	N/A	N/A	N/A
	1y – 6y	45	27	476	40	505	50	36	596
	>6y	65	45	787	50	650	68	46	786

**Table 8:** 75th percentile of CTDIvol and DLP per acquisition for chest and abdomen-pelvis CT examination according to weight, compared with the EDRLs and the values from our previous study CTDIvol: volume computed tomography dose index; DLP: dose length product; CTDIvol in mGy; DLP in mGy·cm; ^†^DLP concerns a single sequence

CT Examination	Weight group	Current study		EDRLs		Authors’ previous study [9]	
		CTDIvol	DLP†	CTDIvol	DLP†	CTDIvol	DLP†
Chest	<5kg	1	18	1.4	35	2	22
	5kg- <15kg	2	33	1.8	50	2	41
	15kg- <30kg	2	42	2.7	70	2	44
	30kg- <50kg	3	74	2.7	115	3	86
	50kg - <80kg	7	226	5.4	200	5	168
Abdomen-pelvis	5kg- <15kg	1	36	3.5	120	2	58
	15kg- <30kg	2	63	5.4	150	2	78
	30kg- <50kg	3	89	7.3	210	3	116
	50kg - <80kg	7	438	13	480	10	425

LDRLs in terms of CTDIvol for head, chest, and abdomen-pelvis CT examinations ranged from 15 to 65 mGy, 1 to 5 mGy, and 1 to 7 mGy, respectively. In terms of DLP, LDRLs for head, chest, and AP CT examinations varied from 211 to 787 mGy·cm, 18 to 226 mGy·cm, and 36 to 438 mGy·cm for the various patient groups, respectively. DLP concerns a single acquisition. CTDIvol values in head CT examinations were substantially higher than those of chest and AP CT examinations due to the high bone density in the skull.

## Discussion

In the current study, we modified our age-based CT protocols in accordance with the new European guidelines RP 185 [7], which suggest specific age groups for head and weight groups for body CT examinations. Following these adjustments, we collected the dosimetric parameters (CTDIvol and DLP) to establish LDRLs for pediatric patients who underwent head, chest, and AP CT examinations according to the newly defined patient groupings.

LDRLs in terms of CTDIvol for head, chest, and AP CT examinations ranged from 15 to 65 mGy, 1 to 5 mGy, and 1 to 7 mGy, respectively. In terms of DLP, LDRLs for head, chest, and AP CT examinations varied from 211 to 787 mGy·cm, 18 to 226 mGy·cm, and 36 to 438 mGy·cm for the various patient groups, respectively. DLP concerns a single acquisition. CTDIvol values in head CT examinations were substantially higher than those of chest and AP CT examinations due to the high bone density in the skull.

Although comparing pediatric DRLs is challenging due to the limited availability of dose data for pediatric patients and the lack of standardization among pediatric CT protocols [7], several recent studies have followed the suggested patient grouping [9-11]. The results of our research revealed that the calculated DRLs were lower than the proposed European, except for head CT examinations for children over six years and chest CT examinations in the 50-80 kg weight category [7]. The previous study used just age-based protocols and included three groups for the CT head, two groups for the CT chest, and two groups for the AP CT [9]. Moving the CT head to the new four age-based groups and the CT chest and AP to the five weight-based categories accordingly revealed such discrepancies. This finding prompts inquiries into whether past evaluated groups followed the protocol or not consistently enough in their practices. Moreover, variations in procedures and equipment upgrades that lack sophisticated technologies like artificial intelligence (AI) could also account for some of the disparities. Furthermore, even though our research was carried out at a level, the presence of numerous skilled radiologists and radiographers, each potentially executing the procedure with minor differences, might have impacted the outcomes and added to the global diversity in DRLs. 

It has been noticed that lower doses were used, probably because radiologists are more at ease interpreting images by using methods like ultrasound simultaneously. On the other hand, in older children needing less image noise due to clinical reasons, higher doses were given. One possible explanation for the elevated radiation dose in this case could be the use of adult scanning standards or insufficient head CT modifications, depending on the cranial size. This highlights the significance of properly modifying treatments for older children based on symptoms.

Our findings were quite similar to the results documented by Almen et al., except for patients over six years old in two instances. Children over six years old in head CT examinations and patients weighing between 50 and 80 kg in chest and AP CT examinations [10] stood out as different from the norm mentioned earlier in the report from Almen et al. 

It's interesting to note that the results highlight the limitations of using large weight or age groups, especially for heavier children. Additionally, alternative measurement systems are suggested, such as efficient diameter (size-specific dose estimation (SSDE)), which improves dose streamlining when the system provides it automatically.

Optimization is crucial for these at-risk subgroups who need attention in their care plan and treatment approach for better outcomes and higher image quality results due to differences in body weight among pediatric patients that necessitate a tailored radiation dose adjustment strategy to enhance imaging accuracy efficiently and effectively. 

Compared to a recent study aiming to establish DRLs for chest computed tomography in pediatric patients as a function of patient size, our results exceeded the proposed LDRLs [12], highlighting the significant variability observed in practice once more. In a study conducted internally, it was observed that by using the new patient grouping methods instead of age-based protocols [9], there was a significant decrease in radiation doses ranging from 0.13% to 61 %. This showcases how precise patient categorization plays a role in optimizing dosage levels effectively. 

Adjustments in CT settings to better suit the patients' size have led to improved radiation control outcomes with body CT scans using weight as a parameter, except for chest CT scans for patients weighing over 50 kg mentioned earlier, due to the evolving nature of our department's protocols transitioning from old to new practices. It is noteworthy to add that exposure characteristics for the head, chest, and AP CT scans varied by age or weight category and were influenced by the manufacturers as well as recommendations from the department's scientific specialists.

In our workplace setting, where radiographers have the authority to modify scanning protocols according to patient needs may have led to variations due to individual judgment calls being made by different staff members. 

In order to address such a problem and minimize any potentially hazardous activities, an automated dose monitoring system will be implemented as part of a clinical review procedure. At the same time, strategies are being updated to deal with maintaining image quality while optimizing dose levels. Another future project will use specialized dose monitoring software to help modify doses according to requirements.

To address such a variance, guidelines could be added to SSDE or customized scanning techniques directed by AI-based dose management systems that seek to provide high-quality diagnostic images without subjecting patients to excessive radiation [13-15]. 

Our study only assessed CTDIvol and DLP values, ignoring subjective evaluations of image quality; hence, we were unable to accurately analyze whether dosage decreases affected diagnosis effectiveness. Even though radiologists remain confident in their findings, lower dosages may raise noise levels, making it more difficult to identify concerns. 

To validate the effectiveness of dose protocols, our upcoming research should cover both quantitative and qualitative assessments of images. Also, our protocol designs didn't fully address the needs (clinical indications). Clinical factors like kilovoltage peak (kVp) and milliampere-seconds (mAs) settings and the length of scans are usually decided based on needs and have a direct impact on the amount of radiation exposure received [16].

In healthcare settings, categorizing patients purely on the basis of age or weight may overlook diagnostic variables and result in a misinterpretation of routine procedures. Therefore, DRLs established without considering the context might not realistically represent how these standards are actually applied in practice.

Balancing radiation dose and image quality for patients can be challenging due to differences in anatomy and physiology compared to adults. Despite similarities in certain anatomical features between children and adults, weight-based grouping seems to be more effective in optimizing doses than age-based protocols, as physiological development may not always align with chronological age, according to our findings.

To sum up, it is crucial to take into account both patient size and the clinical indication when developing future initiatives and advancements in the field of reinforcement learning (DRL) in order to guarantee its clinical relevance and practical usability in real-world scenarios, particularly for the pediatric population.

## Conclusions

The study described the transition from age-based to weight-based CT protocols for body CT examinations and the implementation of LDRLs using the updated protocols. The results showed that the revised age- and weight-based protocols led to lower radiation doses compared to the previous age-based CT protocols, suggesting that weight-based protocols are more suitable for body CT examinations. Our team will kick off with SSDE as one of the forthcoming study initiatives as soon as the new CT unit arrives, due to the automatic procedure, which is expected to lead to safe results. The installation of dosage monitoring software in our department will also result in automated dose monitoring and the detection of improper activities. While the locally derived DRLs were generally lower than the EDRLs, additional optimization is needed for head CT examinations in patients over six years of age and for chest CT examinations in patients weighing more than 50 kg. As part of our next actions, we will disclose updated exposure factors as soon as brand-new CT equipment arrives, in compliance with the clinical audit department's recommendations. Since different medical reasons require different image quality, even for the same body part (like head injuries, tumors, or headaches), we should also focus on improving DRLs and checking image quality based on the specific medical reasons.
